# Juvenile Hippocampal CA2 Region Expresses Aggrecan

**DOI:** 10.3389/fnana.2017.00041

**Published:** 2017-05-10

**Authors:** Asako Noguchi, Nobuyoshi Matsumoto, Shota Morikawa, Hideki Tamura, Yuji Ikegaya

**Affiliations:** ^1^Graduate School of Pharmaceutical Sciences, The University of TokyoTokyo, Japan; ^2^Life Science Tokyo Advanced Research Center (L-StaR), School of Pharmacy and Pharmaceutical Sciences, Hoshi UniversityTokyo, Japan; ^3^Center for Information and Neural Networks, National Institute of Information and Communications TechnologyOsaka, Japan

**Keywords:** perineuronal nets, CA2, postnatal development, anterior-posterior, immunohistochemistry

## Abstract

Perineuronal nets (PNNs) are distributed primarily around inhibitory interneurons in the hippocampus, such as parvalbumin-positive interneurons. PNNs are also present around excitatory neurons in some brain regions and prevent plasticity in these neurons. A recent study demonstrated that PNNs also exist around mouse hippocampal pyramidal cells, which are the principle type of excitatory neurons, in the CA2 subregion and modulate the excitability and plasticity of these neurons. However, the development of PNNs in the CA2 region during postnatal maturation was not fully investigated. This study found that a main component of PNNs, aggrecan, existed in the pyramidal cell layer of the putative CA2 subarea prior to the appearance of the CA2 region, which was defined by the CA2 marker protein regulator of G protein signaling 14 (RGS14). We also found that aggrecan immunoreactivity was more evident in the anterior sections of the CA2 area than the posterior sections, which suggests that the function of CA2 PNNs varies along the anterior-posterior axis.

## Introduction

Perineuronal nets (PNNs) are extracellular macromolecules that consist of several components, such as chondroitin sulfate proteoglycans, hyaluronan and tenascin-R. The chondroitin sulfate proteoglycan aggrecan is almost exclusively present in PNNs, and it is often used as a marker of PNNs (Matthews et al., 2002). PNNs generally envelop the surface of inhibitory interneurons in the hippocampus (Celio and Chiquet-Ehrismann, 1993; Celio et al., 1998) and enhance their ability to restrict synaptic plasticity (Pizzorusso et al., 2002; Gogolla et al., 2009; Orlando et al., 2012; Balmer, 2016). However, a recent study demonstrated that PNNs also exist around the excitatory pyramidal neurons in the CA2 subregion in the mouse hippocampus and prevent plasticity at these excitatory synapses (Carstens et al., 2016). These observations suggest that PNNs increase the threshold of synaptic plasticity of the CA2 region (Zhao et al., 2007; Chevaleyre and Siegelbaum, 2010). One possible mechanism underlying this modulation is the buffering of cations, including calcium ions, through negatively charged proteoglycans in PNNs (Bruckner et al., 1993).

No previous study investigated the detailed distribution of PNNs, especially in the CA2 area. More specifically, the distribution of PNNs, primarily in inhibitory interneurons, differs between the dorsal and ventral hippocampus (Yamada and Jinno, 2013), but differences along the anterior-posterior axis were not investigated in the CA2 pyramidal cell layer. Therefore, we scrutinized the immunohistochemical signal for aggrecan along the anterior-posterior axis and found that aggrecan was more abundant in the anterior CA2 region than the posterior CA2 region. We also traced the postnatal development of PNNs, which was not examined using PNN proteoglycan markers, despite the lack of synaptic plasticity in the CA2 region, even early in development (Zhao et al., 2007). We found that aggrecan immunoreactivity was evident in the putative CA2 region at postnatal day (P) 5.

## Materials and Methods

### Animal Ethics

Animal experiments were performed with the approval of the animal experiment ethics committee at the University of Tokyo (approval number: 24–10) and in accordance with the University of Tokyo guidelines for the care and use of laboratory animals. The experimental protocols were performed in accordance with the Fundamental Guidelines for the Proper Conduct of Animal Experiments and Related Activities in Academic Research Institutions (Ministry of Education, Culture, Sports, Science and Technology, Notice No. 71 of 2006), the Standards for Breeding and Housing of and Pain Alleviation for Experimental Animals (Ministry of the Environment, Notice No. 88 of 2006) and the Guidelines on the Method of Animal Disposal (Prime Minister’s Office, Notice No. 40 of 1995).

### Histology

Three P7, four P14, three P21, or 10 young adult (4–8 weeks old) male C57BL/6J mice were anesthetized via intraperitoneal administration of 150 mg/ml urethane dissolved in saline. P3-to-6-day-old mice were anesthetized in ice (*n* = 3 mice for each age). Anesthesia was confirmed via the lack of reflex responses to tail and toe pinch. Mice were transcardially perfused with ice-cold phosphate-buffered saline (PBS) followed by 4% paraformaldehyde in PBS, and the brains were removed. Brains were post-fixed in 4% paraformaldehyde overnight, washed with PBS three times for 10 min each and coronally sectioned at a thickness of 100 μm using a vibratome from the anterior region to the posterior region. The most anterior section for each brain was considered when the cross-sectional area of the hippocampus exceeded 0.5 mm^2^. The anterior-posterior locations of slices were expressed relative to the most anterior section (0 μm). Five slices (or four slices for P6 and three slices for P3-to-5-day-old mice) were cut at a thickness of 100 μm and collected every 400 μm after the 0-μm section. These sections nearly covered the entire dorsal hippocampal CA2 area at each age. Sections were blocked with 10% goat serum and 3% Triton X-100 in PBS for 60 min and incubated with a mouse primary antibody against regulator of G protein signaling 14 (RGS14; 1:500, 75–170, NeuroMab, CA, USA) and a rabbit primary antibody against aggrecan (1:2000, AB1031, Merck Millipore, Billerica, MA, USA) or a mouse primary antibody against neurocan (1:1000, ab26003, Abcam, Cambridge, UK) for 16 h. Sections were washed three times for 10 min with PBS and incubated with Alexa Fluor 488-conjugated goat secondary antibody against mouse IgG (1:500, A11001, Invitrogen, MA, USA), Alexa Fluor 594-conjugated goat secondary antibody against rabbit IgG (1:500, A11037, Invitrogen, MA, USA), and blue fluorescent Neuro Trace (1:500, N21479, Thermo Fisher Scientific, MA, USA) for 6 h. Brains for WFA immunostaining were post-fixed overnight at 4°C in 4% paraformaldehyde in PBS and transferred to 30% sucrose in PBS for 48 h. Brains were cut into 30 μm coronal sections using a cryostat (CM1860, Leica Co., Wetzlar, Germany). Free-floating sections were blocked with 5% bovine serum albumin and 0.3% Triton X-100 in PBS for 1 h at room temperature. Sections were incubated overnight at 4°C in biotin-conjugated WFA (1:3000; BA-3101, EY Laboratories, Inc., San Mateo, CA, USA) and a rabbit primary antibody against aggrecan (1:2000, AB1031, Merck Millipore, Billerica, MA, USA), followed by Alexa 594-conjugated streptavidin (0.67 μg/ml; S11227, Thermo Fisher Scientific), Alexa Fluor 488-conjugated secondary donkey antibody against rabbit IgG (1:1000, A21206, Invitrogen, MA, USA), and blue fluorescent Neuro Trace (1:500, N21479, Thermo Fisher Scientific, MA, USA). Labeled sections were mounted onto microscope slides using Prolong Diamond Antifade Reagent (Thermo Fisher Scientific). The colors of WFA and aggrecan staining were swapped in Figure 12 to be consistent with the other figures. Green and red represent WFA and aggrecan, respectively.

**Figure 1:**
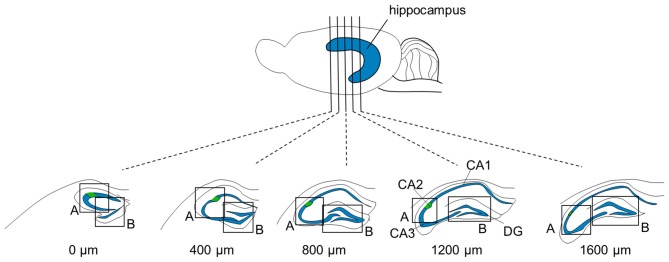
**Focused areas of brain sections for immunostaining.** Five (or four for P6 and three for P3-to-5-day-old mice) 100-μm-thick sections were prepared at intervals of 400 μm from one brain. The measures indicate the distances from the first section in which the rostral tip of the hippocampus appeared. Sections were immunolabeled for aggrecan and RGS14 and counterstained with blue-fluorescent Nissl. Regions, including the CA2 area and dentate gyrus (DG), indicated by boxes (A) and (B), respectively, were confocally imaged and *Z*-stacked.

**Figure 2:**
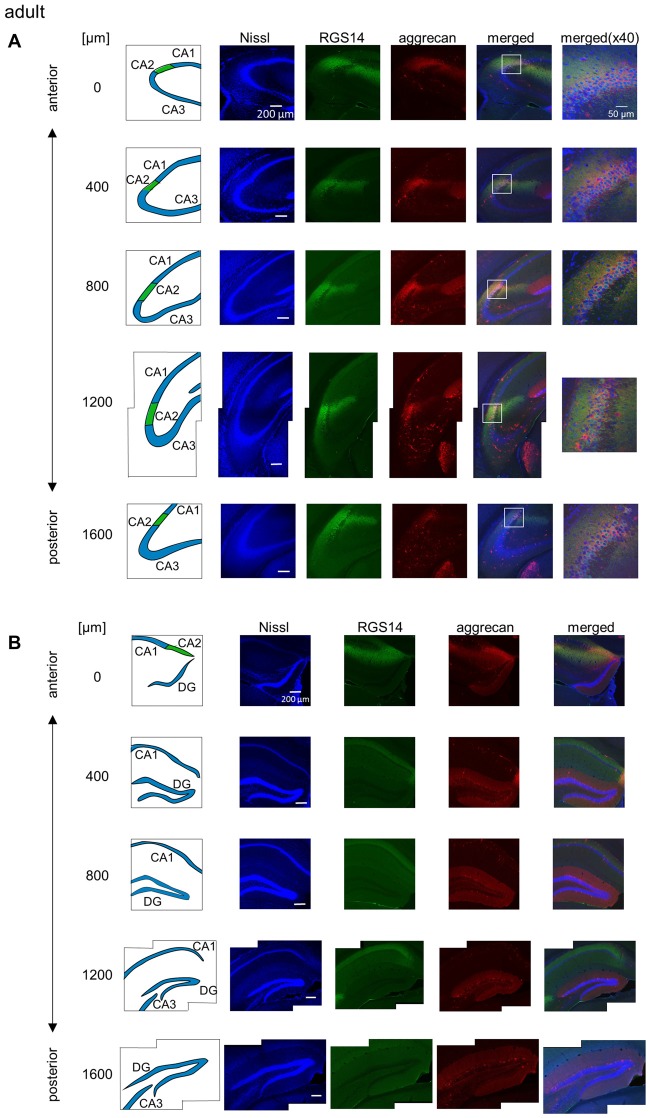
**Representative photographs of regulator of G protein signaling 14 (RGS14) and aggrecan immunoreactivity in the hippocampus from an adult mouse along the anterior-posterior axis. (A)** In each row, the left column depicts the hippocampal substructure in the areas imaged in the four middle columns in which Nissl (blue), anti-RGS14 (green) and anti-aggrecan (red) were confocally imaged and superimposed. The boxed area including the CA2 subregion is magnified in the right column. Different rows display photographs obtained from different anterior-posterior levels, which are indicated as the distance (μm) from the most anterior section. **(B)** Same as **(A)**, but for areas including the DG. High-magnification images were not captured for the DG. Experiments were repeated for all 10 animals of the same age, and the same results were obtained.

**Figure 3:**
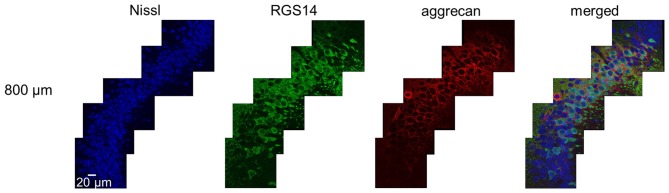
**Representative high-magnification photographs of RGS14 and aggrecan immunoreactivity in the hippocampal CA2 area from an adult mouse along the anterior-posterior axis.** High-magnification (100×) images of the hippocampal CA2 area in which Nissl (blue), anti-RGS14 (green) and anti-aggrecan (red) were confocally imaged and superimposed. The section shown are the middle of five sections along the anterior-posterior axis. The other four sections showed the same distribution of RGS14 and aggrecan. Experiments were repeated for all 10 animals of the same age, with the same results.

**Figure 4:**
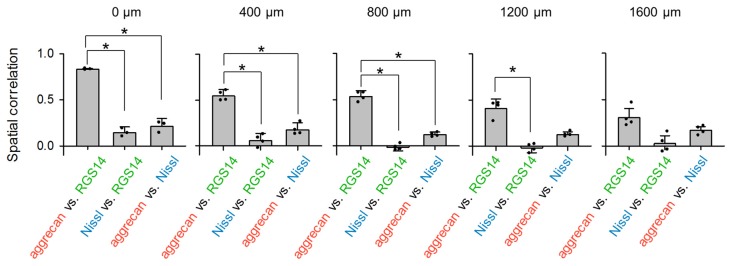
**Aggrecan immunoreactivity in CA2 pyramidal cells is more evident in the anterior hippocampus of the adult mouse hippocampus.** The numbers above the panels indicate the distance from the most anterior section. For each anterior-posterior level, we calculated the spatial correlations between the fluorescence intensities of aggrecan and RGS14 (left), Nissl and RGS14 (middle) and aggrecan and Nissl (right). In the sections at 0, 400 and 800 μm, the correlations between aggrecan and RGS14 ranged between 0.47 and 0.84 and were significantly higher than for the other pairs, which indicates that the aggrecan-positive areas overlapped significantly with the RGS14-positive areas (*P < 0.05, one-way analysis of variance (ANOVA) followed by Bonferroni’s *post hoc* test). Significant overlap was not observed in posterior sections at 1200 and 1600 μm. Error bars are the standard deviation (SD) of 3–6 sections from six mice.

**Figure 5:**
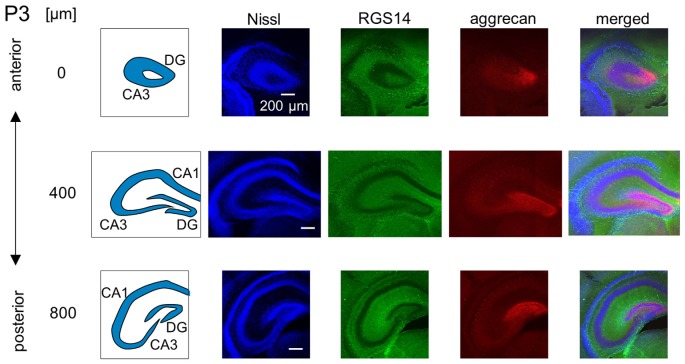
**Representative confocal photographs of RGS14 and aggrecan immunoreactivity in the hippocampus of a P3 mouse.** The schematics in the left column depict the hippocampal substructure. Fluorescence images of Nissl (blue), anti-RGS14 (green), anti-aggrecan (red) and their superimposition are shown in the four right columns. Different rows indicate photographs obtained from different anterior-poster levels. Aggrecan immunoreactivity was observed primarily in the DG. Experiments were repeated for three animals of the same age, with the same results.

**Figure 6:**
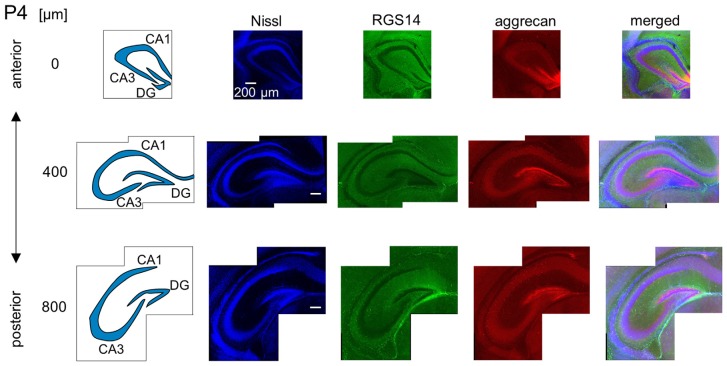
**Representative images of a P4 mouse.** The schematics in the left column depict the hippocampal substructure. Fluorescence images of Nissl (blue), anti-RGS14 (green), anti-aggrecan (red) and their superimposition are shown in the four right columns. Aggrecan immunoreactivity was observed primarily in the DG, but it was also detectable in the entire pyramidal cell layer. Experiments were repeated for three animals of the same age, with the same results.

**Figure 7:**
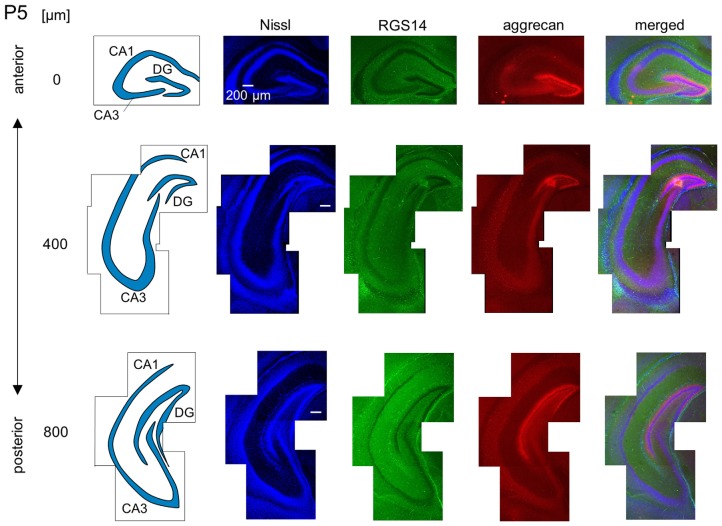
**Representative images of a P5 mouse.** The schematics in the left column depict the hippocampal substructure. Fluorescence images of Nissl (blue), anti-RGS14 (green), anti-aggrecan (red) and their superimposition are shown in the right columns. In the anterior hippocampus, the putative CA2 area began to express aggrecan, but not RGS14. The entire DG also expressed aggrecan. Experiments were repeated for three animals of the same age, with the same results.

**Figure 8:**
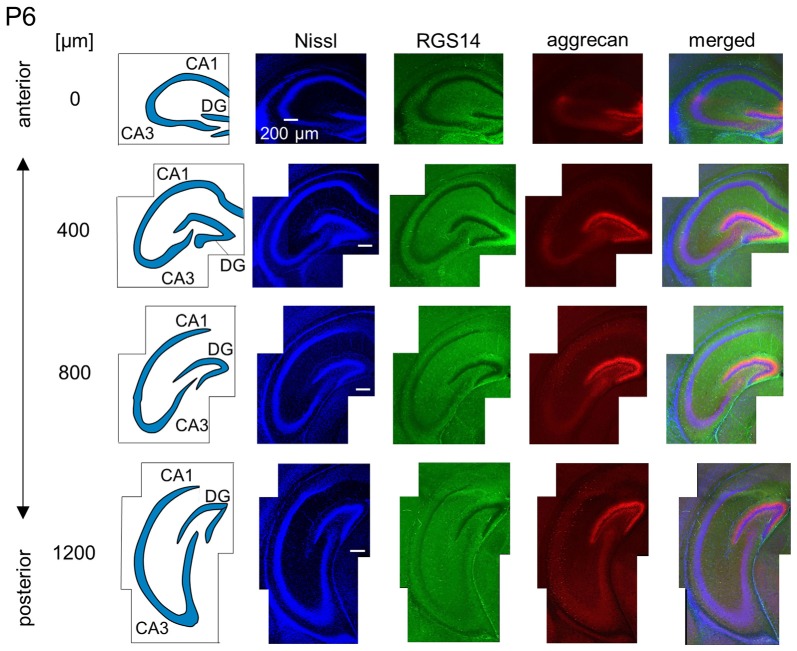
**Representative images of a P6 mouse.** The schematics in the left column depict the hippocampal substructure. Fluorescence images of Nissl (blue), anti-RGS14 (green), anti-aggrecan (red) and their superimposition are shown in the right columns. Aggrecan immunoreactivity in the putative CA2 area became stronger in the anterior hippocampus relative to the P5 hippocampi. It was also slightly detected in the sections at 400 and 800 μm, and RGS14 was absent from the putative CA2 area. Aggrecan was expressed in the entire DG. Experiments were repeated for three animals of the same age, with the same results.

**Figure 9:**
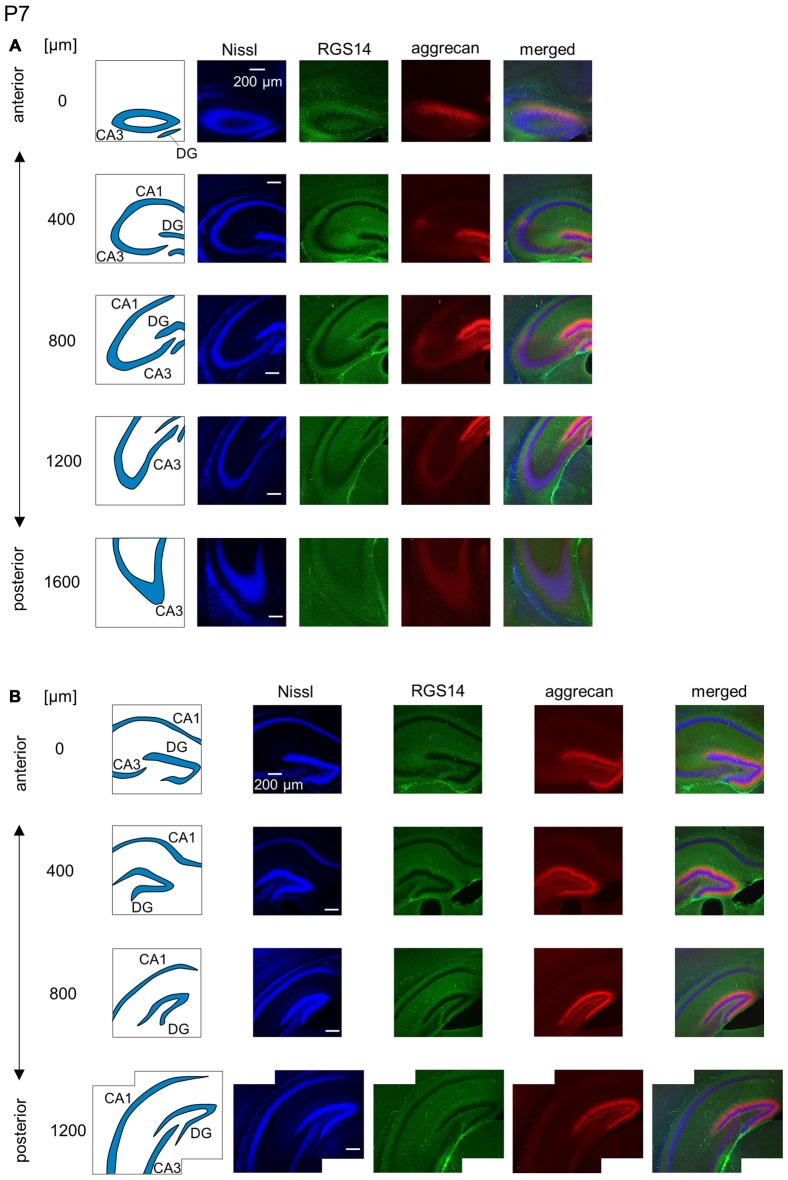
**Representative images of the hippocampus in a P7 mouse. (A)** The schematics in the left column depict the hippocampal substructure. Fluorescence images of Nissl (blue), anti-RGS14 (green), anti-aggrecan (red) and their superimposition are shown in the four right columns. RGS14 and aggrecan immunoreactivity exhibited a pattern similar to the P6 hippocampus, but RGS14 was faintly detectable in the CA2 area. **(B)** The same as in **(A)**, but for the DG. Aggrecan was expressed in the entire DG. Experiments were repeated for three animals of the same age, with the same results.

**Figure 10:**
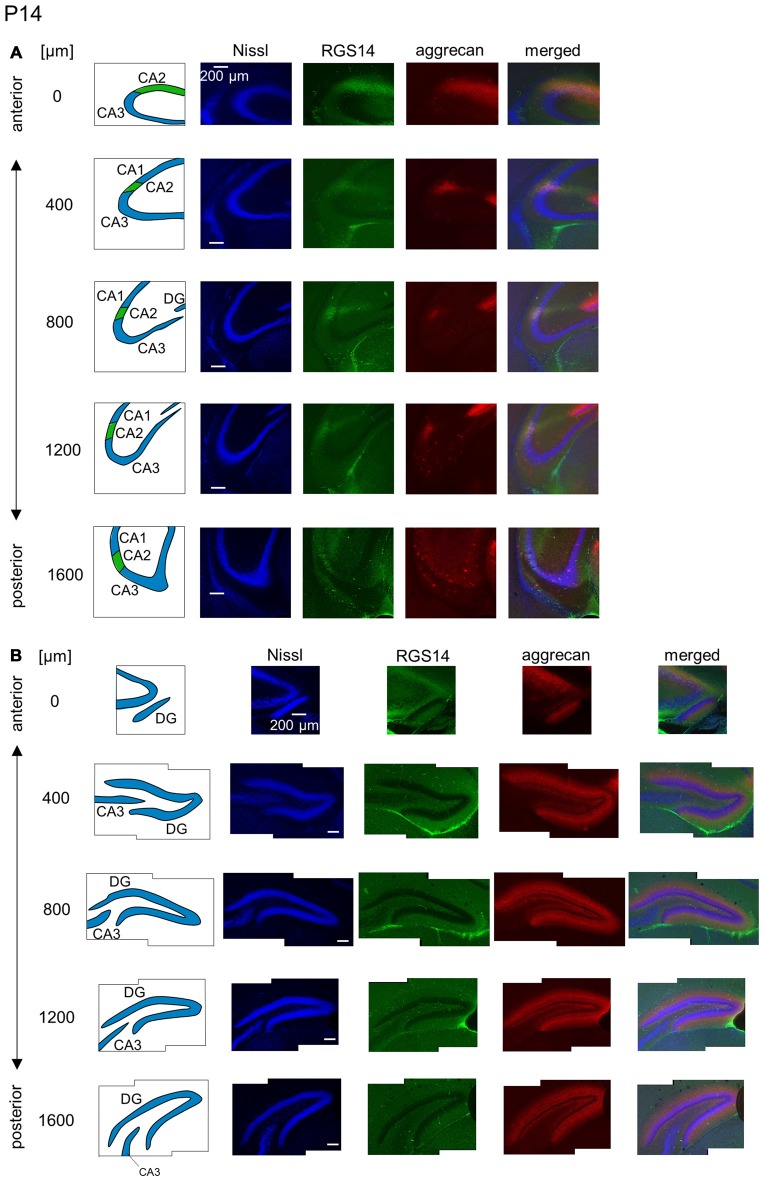
**Representative images of the hippocampus in a P14 mouse. (A)** The schematics in the left column depict the hippocampal substructure. Fluorescence images of Nissl (blue), anti-RGS14 (green), anti-aggrecan (red) and their superimposition are shown in the four right columns. In all five sections, the CA2 region was clearly defined by RGS14 expression. Aggrecan around the CA2 pyramidal cells was present in four anterior sections. **(B)** The same as in **(A)** but for the DG, in which aggrecan immunoreactivity was present but became less strong than in younger animals. Experiments were repeated for four animals of the same age, with the same results.

**Figure 11:**
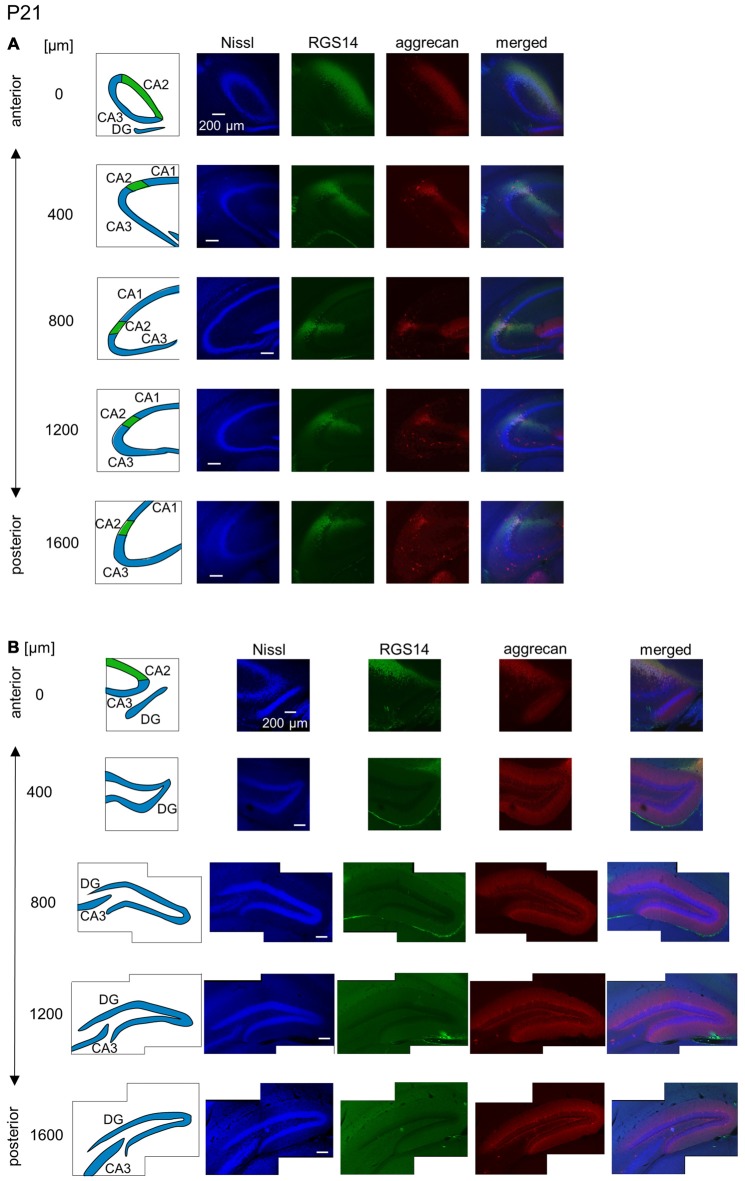
**Representative images of the hippocampus in a P21 mouse.** The panels are the same as Figure 9 but at P21. Immunoreactivity exhibited patterns similar to the adult hippocampus. Experiments were repeated for three animals of the same age, with the same results.

**Figure 12:**
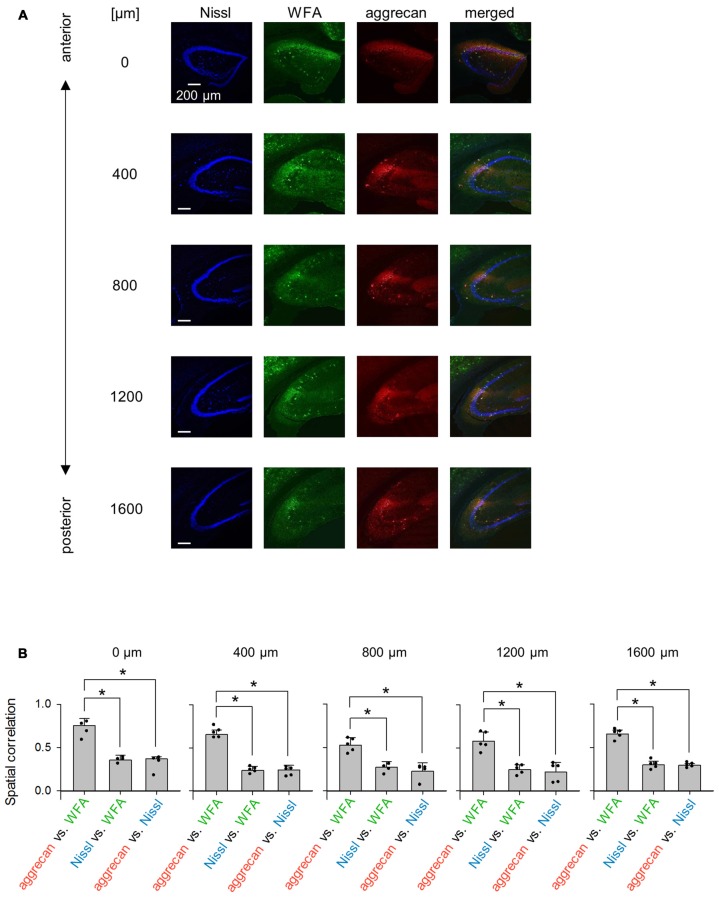
**Representative images of WFA and aggrecan immunoreactivity in the hippocampus including the CA2 area from an adult mouse along the anterior-posterior axis. (A)** Fluorescence images of Nissl (blue), WFA (green), anti-aggrecan (red) and their superimposition are shown. Different rows indicate photographs obtained from different anterior-poster levels. Experiments were repeated for five animals of the same age, with the same results. **(B)** The numbers above the panels indicate the distance from the most anterior section. For each anterior-posterior level, we calculated the spatial correlations between the fluorescence intensities of aggrecan and WFA (left), Nissl and WFA (middle) and aggrecan and Nissl (right). In the all five sections, the correlations between aggrecan and WFA ranged between 0.43 and 0.80 and were significantly higher than the other pairs, which indicates that the aggrecan-positive areas significantly overlapped with the WFA-positive areas (*P < 0.05, one-way ANOVA followed by Bonferroni’s *post hoc* test). Error bars are the SD of 4–5 sections from five mice.

### Confocal Imaging

Six images (1024 × 1024 pixels, 16-bit intensity) for each region of interest were acquired at a *Z*-interval of 0.5 μm using an FV1200 confocal microscope (Olympus, Tokyo, Japan) equipped with 10×, 40×, or 100× objectives and *Z-stacked* using ImageJ software (National Institutes of Health, MD, USA).

### Statistics

The data were analyzed using MATLAB (MathWorks, Natick, MA, USA). The summarized data are reported as the mean ± SD. *P* < 0.05 was considered statistically significant. We split the colors of each image into RGB mode to assess the spatial correlation between aggrecan and RGS14, Nissl and RGS14, or Nissl and aggrecan and obtained the color intensity of each pixel of the three-color images. We created three matrices, including such elements as the pixel ID numbers, and calculated Pearson’s correlations between two of the three matrices.

## Results

We sliced the brains of 7-to-8-week-old mice into five 100-μm-thick sections every 400 μm to investigate the distribution of PNNs around the dorsal hippocampal CA2 pyramidal neurons along the anterior-posterior axis (Figure 1) and performed immunostaining against aggrecan, a component of PNNs, and the CA2 marker RGS14 (Kohara et al., 2014). Brains were counterstained with blue fluorescent Neuro Trace (blue fluorescent Nissl). We detected aggrecan-positive areas in the CA2 region of all 10 mice tested using a 10× objective lens (Figure 2A). We confirmed that aggrecan immunoreactivity surrounded RGS14-positive CA2 pyramidal neurons under higher magnification (100×; Figure 3), which is consistent with a previous study (Carstens et al., 2016). A slightly positive aggrecan signal was found at the border between the granule cell layer and the molecular layer in the dentate gyrus (DG; Figure 2B).

We calculated the spatial correlation between pairs of three fluorescence channels to quantify the overlap between aggrecan-positive and RGS14-positive (i.e., CA2) areas: red and green for aggrecan and RGS14, respectively; blue and green for Nissl and RGS14, respectively; and blue and red for Nissl and aggrecan, respectively (Figure 4). The results differed across the anterior-posterior axis. The spatial correlation between aggrecan and RGS14 for the three anterior slices was significantly higher than the other two pairs (0 μm: *P* = 2.37 × 10^−6^, *F*_(2,6)_ = 5.14, *n* = 3; 400 μm: *P* = 3.49 × 10^−6^, *F*_(2,9)_ = 4.26, *n* = 4; 800 μm: *P* = 1.85 × 10^−8^, *F*_(2,9)_ = 4.26, *n* = 4; Bonferroni’s *post hoc* test for pairwise comparisons after one-way analysis of variance (ANOVA)). The second posterior section exhibited a significant difference only between one pair (1200 μm: *P* = 1.30 × 10^−5^, *F*_(2,9)_ = 4.26, *P* (aggrecan and RGS14 vs. Nissl and RGS14) = 2.51 × 10^−3^, *P* (aggrecan and RGS14 vs. aggrecan and Nissl) = 0.05, *P* (Nissl and RGS14 vs. aggrecan and Nissl) = 0.06, *n* = 4; Bonferroni’s *post hoc* test for pairwise comparisons after one-way ANOVA).The most posterior slice exhibited no significant differences between the three pairs (1600 μm: *P* = 5.15 × 10^−3^, *F*_(2,9)_ = 5.14, *P* (aggrecan and RGS14 vs. Nissl and RGS14) = 0.07, *P* (aggrecan and RGS14 vs. aggrecan and Nissl) = 0.18, *P* (Nissl and RGS14 vs. aggrecan and Nissl) = 0.49, *n* = 4; Bonferroni’s *post hoc* test for pairwise comparisons after one-way ANOVA). Therefore, the spatial congruity between aggrecan and the CA2 region was higher in the anterior CA2 area than the posterior CA2 area.

We investigated the postnatal development of the expression of aggrecan. We performed the same immunohistochemical staining of hippocampi obtained from P3, 4, 5, 6, 7, 14 and 21 mice. No specific immunopositive areas existed for aggrecan or RGS14 at P3. However, the immunosignal for aggrecan was detected weakly in the entire pyramidal cell layer, and it was denser in the CA3c area more proximal to the DG (Figure 5). Notably, a strong immunosignal was observed in the DG. The expression patterns of aggrecan in P4 mice were similar to P3 mice (Figure 6).

We observed a specific accumulation of aggrecan within the pyramidal cell layer in P5 mice (Figure 7). Aggrecan was observed in the putative CA2 area in the most anterior section (0 μm) of P5 mice (Figure 7). This segregation primarily occurred because the border between the aggrecan-positive area and the aggrecan-negative CA1, but not CA3, region was now evident. However, RGS14 was not expressed at this age, and we were unable to identify the true area corresponding to the CA2 region defined by RGS14. Aggrecan expression was most strongly expressed in the DG (Figure 7). Aggrecan immunoreactivity at P6 was similar to P5, but it was more evident in the putative CA2 area because the border between the aggrecan-positive CA2-like area and the aggrecan-negative CA3 region became easily detectable (Figure 8).

The aggrecan-positive CA2-like area at P7 extended to three anterior sections, and RGS14 became faintly evident in the CA2 area (Figure 9A). Aggrecan expression remained highest in the DG (Figure 9B). The RGS14 signal at P14 was evident in all sections, and we confirmed that the aggrecan-positive area corresponded to the CA2 region (Figure 10A). The aggrecan signal was absent from the most posterior sections. The aggrecan signal in the DG at P14 was weaker than P7 (Figure 10B). Immunostaining at P21 exhibited a pattern similar to the adult hippocampus (Figure 11).

In summary, aggrecan was expressed in the pyramidal cell layer at P3, but its expression was not specific to the CA2 area. Specific aggrecan expression was first detected at P5 in the most anterior section, and it became clearer until P21. We also observed that aggrecan expression was strongest in the DG of immature mice and gradually became less intense during development.

**Figure 13:**
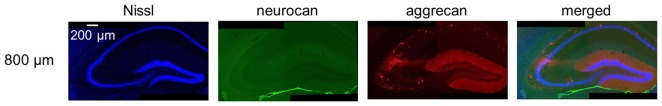
**Representative images of neurocan and aggrecan immunoreactivity in the hippocampus including the CA2 area from an adult mouse.** The panels are the same as the image of 800 μm-section in Figure 12 but for neurocan instead of WFA. Neurocan was not expressed as previously reported. Experiments were repeated for three animals of the same age, with the same results.

We repeated similar immunohistochemical staining using WFA and an anti-neurocan antibody to compare other putative PNN markers to aggrecan (Figures 12A, 13). We calculated the spatial correlations to quantify the overlap between aggrecan-positive and WFA-positive areas. The spatial correlation between aggrecan and WFA was significantly higher than the other two pairs for all five section levels (0 μm: *P* = 1.17 × 10^−4^, *F*_(2,6)_ = 4.26, *n* = 4; 400 μm: *P* = 8.23 × 10^−9^, *F*_2 9_ = 3.89, *n* = 5; 800 μm: *P* = 9.04 ×10 ^−5^, *F*_(2,9)_ = 3.89, *n* = 5; 1,200 μm: *P* = 1.12 × 10^−4^, *F*_(2,9)_ = 3.89, *n* = 5; 1,600 μm: *P* = 3.32 × 10^−8^, *F*_(2,9)_ = 3.89, *n* = 5; Bonferroni’s *post hoc* test for pairwise comparisons after one-way ANOVA). Therefore, aggrecan-positive and WFA-positive areas overlapped in adult mice. In contrast, neurocan expression was hardly observed in the hippocampal area, which is consistent with a previous report (Matsui et al., 2002).

## Discussion

**Figure 14:**
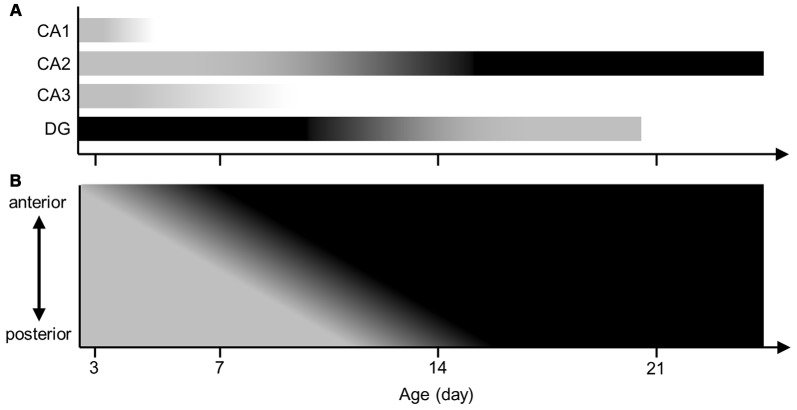
**Illustrated summary of aggrecan expression patterns in hippocampal pyramidal cell layer and DG. (A)** The expression levels of aggrecan in the CA1, CA2 and CA3 areas and the DG are presented in a gray scale during postnatal development. Darker colors represent higher amounts of aggrecan. **(B)** The expression levels of aggrecan in the CA2 along the hippocampal anterior-posterior axis are illustrated along postnatal development.

Several studies reported that the embryonic brain already expressed aggrecan in animals other than mice (Milev et al., 1998; Schwartz and Domowicz, 2004). Another study demonstrated that aggrecan mRNA was present in the visual cortex of P3 mice (Carulli et al., 2010). However, aggrecan in the immature hippocampus was not investigated. The present study provided a series of immunohistochemical images of aggrecan during the postnatal development of the mouse hippocampus. Therefore, we propose several hypotheses: (i) PNNs exist nearly exclusively around CA2 pyramidal cells among the excitatory neurons of the adult hippocampus; (ii) the anterior CA2 area expresses PNNs more heavily than the posterior CA2 region; (iii) aggrecan is present in the entire pyramidal cell layer at P3, in the putative CA2 region after P5, and mature until P21; and (iv) RGS14 defines the CA2 region only after P7 (Figure 14).

Development of the CA2 region was investigated using another marker protein, PCP4 (San Antonio et al., 2014). PCP4 immunoreactivity appeared diffusely at P4–5 and gradually increased by P7. The PCP4-positive region becomes more restricted to the CA2 region by approximately P10 and matures until P21 (San Antonio et al., 2014). RGS14 (Evans et al., 2014) is first expressed in the P7 hippocampus. Therefore, aggrecan may be used as an early marker of the CA2 region, perhaps in the premature CA2 region.

Our observation of PNNs in the mature hippocampal CA2 area is consistent with a recent study (Carstens et al., 2016), which further demonstrated that PNNs around CA2 pyramidal cells suppressed the plasticity of their excitatory synapses. How these PNNs inhibit neuronal excitation is not fully known. PNNs contain polyanionic components and proteoglycans, which buffer cations (Bruckner et al., 1993). This function was investigated primarily in PNNs around inhibitory neurons, but we hypothesized that PNNs around excitatory neurons in the hippocampal CA2 area also perform this function because excitatory PNNs contain negatively charged proteoglycans, such as aggrecan, similarly to inhibitory PNNs.

One study investigated the postnatal development of PNNs in the hippocampus using immunostaining against WFA lectin (Giamanco et al., 2010) and demonstrated that PNNs appeared at P14 and matured until P21 in the CA2 pyramidal cell layer (Horii-Hayashi et al., 2015). This time course appears inconsistent with our data. The discrepancy may result from the use of different PNN markers. WFA lectin recognizes glycans, and aggrecan antibodies recognize the core protein of the proteoglycan. These results suggest that the aggrecan in the CA2 region in mice younger than P7 does not bind glycan, and it is not detectable using WFA lectin. We also demonstrated that aggrecan and WFA signals in adulthood highly overlapped in the hippocampal CA2 area. However, another major proteoglycan, neurocan, was not detected in the hippocampus. These results suggest that the proteoglycans of PNNs exhibit a specific composition in the hippocampal CA2 region.

We also observed regions other than the CA2 region in the hippocampal formation. Aggrecan immunosignal first appeared around the granule cell layer of the DG at P3 and gradually spread toward the pyramidal cell layer, particularly in the CA3 region proximal to the DG. An aggrecan immunosignal was present in the entire pyramidal cell layer at P4, irrespective of the CA1, CA2 and CA3 regions. Thereafter, the border between the aggrecan-positive CA2-like area and the aggrecan-negative CA1 region became evident at P5 in the most anterior hippocampus, and the border between the aggrecan-positive area and the aggrecan-negative CA3 region was easily detectable at P6. This observation suggests that aggrecan disappeared earlier in the CA1 region than the CA3 region, and aggrecan expression in the CA2 region persisted until adulthood. Aggrecan expression in the DG was also denser in younger mice compared to mature mice. Aggrecan expression in the adult DG was restricted to the border between the granule cell layer and the molecular layer, which suggests its presence primarily in interneurons. Therefore, aggrecan was more heavily expressed around immature DG granule cells, in contrast to CA2 neurons. Whether electrophysiological properties differ between immature and mature granule cells would be an intriguing discovery. For example, young granule cells exhibit a higher input resistance and lower spike threshold than adult granule cells (Schmidt-Hieber et al., 2004). Giant bursts of inhibitory currents only occur in immature DG at the circuit level (Hollrigel et al., 1998). Newly generated granule cells exhibit greater synaptic plasticity than mature granule cells in the adult hippocampus (Schmidt-Hieber et al., 2004; Ge et al., 2007). These differences may arise partially from the expression level of aggrecan.

The anterior-to-posterior difference in the CA2 region was not investigated intensively, likely because this region is considerably smaller than the CA1 or CA3 region. Therefore, our findings on the different distribution of aggrecan immunosignal across the anterior-posterior axis may provide new information on the properties of the CA2 area. We found that aggrecan was less enriched in the posterior CA2 area in adulthood and development. Aggrecan is expressed in an activity-dependent manner (Lander et al., 1997), and neuronal activity may be higher in the anterior CA2 area than the posterior CA2 region. The axonal connectivity between the hippocampus and with cortical/subcortical structures is a gradient along the longitudinal axis of the hippocampus (Amaral and Witter, 1989; Kjelstrup et al., 2008). A similar gradient may also exist along the anterior-posterior axis. For example, hyperpolarization-activated cyclic nucleotide-gated channels (HCN) and *N*-methyl-D-aspartate (NMDA) receptors exist more heavily in the dorsal hippocampus (Strange et al., 2014). Therefore, the distributions of these channels and receptors may be related to the properties of aggrecan or PNNs, and the abundance of NMDA receptors in the anterior hippocampus may contribute to higher aggrecan expression in an activity-dependent manner. Higher expression of HCN channels may result from a richer distribution of PNNs in the anterior hippocampus via their inhibitory function. Further investigations are needed to determine whether the CA2 synaptic plasticity and its developmental changes differ depending on the location along the anterior-posterior axis and whether the same developmental changes occur in the ventral hippocampus.

## Author Contributions

HT and YI designed the research. AN, NM and SM performed the research. AN and NM analyzed the data. AN, NM, HT and YI wrote the manuscript.

## Funding

This work was supported by a Japan Society for the Promotion of Science Grant-in-Aid for Science Research A (grant no. 26250003) and a Grant-in-Aid for Science Research on Innovative Areas “Mental Time” (grant no. 25119004).

## Conflict of Interest Statement

The authors declare that the research was conducted in the absence of any commercial or financial relationships that could be construed as a potential conflict of interest.
